# Feasibility of Neurospective: A Multi-Domain Cognitive Screening Platform with Gamified Tasks for Early Detection

**DOI:** 10.1093/geroni/igaf122.3591

**Published:** 2025-12-31

**Authors:** Lenora Smith

**Affiliations:** University of Alabama in Huntsville, Huntsville, Alabama, United States

## Abstract

**Background:**

Early detection of cognitive decline requires accessible tools that assess multiple domains. Neurospective is a mobile gamified platform to assess a wide range of cognitive functions through short digital games intended for large-scale community-based use.

**Objectives:**

To evaluate feasibility and usability of a comprehensive mobile cognitive battery incorporating both traditional neurocognitive tasks and novel gamified paradigms, with plans for ML-based scoring.

**Methods:**

The current prototype consists of 24 mobile tasks spanning executive function, attention, memory, psychomotor speed, and visuospatial processing. Stroop serves as the control task. MoCA-aligned tasks include naming animals, a letter-tap inhibition task, serial subtraction, connect-the-dots, and cube drawing. Additional tasks include four versions of memory match, animal and shape counting, naming colors and shapes, digital bingo, motion detection, whack-a-mole, pattern recognition, a block game, and a puzzle game. All tasks were built for compatibility with iOS and Android, and user interactions are logged for duration, touch precision, and error rates. Launch to initial users is planned within the month, with gameplay data sent for machine learning model development.

**Results:**

Pre-launch testing shows platform stability and high task engagement. Users report ease of navigation and positive feedback on task enjoyment. Formal usability data collection is forthcoming.

**Conclusions:**

Neurospective appears feasible as a scalable, user-friendly screening tool integrating both validated and innovative cognitive tasks. The breadth of domains and planned ML scoring may allow for more nuanced detection of cognitive change, positioning Neurospective as a next-generation tool for early cognitive assessment.

